# Significance of hypoxia-inducible factor-1α expression with atrial fibrosis in rats induced with isoproterenol

**DOI:** 10.3892/etm.2014.1989

**Published:** 2014-09-24

**Authors:** FANGJU SU, WEIZE ZHANG, YONGQING CHEN, LING MA, HANPING ZHANG, FEI WANG

**Affiliations:** Department of Cardiology, Lanzhou General Hospital, People’s Liberation Army, Lanzhou, Gansu 730050, P.R. China

**Keywords:** hypoxia inducible factor-1α, atrial, fibrosis, transforming growth factor-β_1_, matrix metalloproteinase-9

## Abstract

Atrial interstitial fibrosis plays a dual role in inducing and maintaining atrial fibrillation (AF). Hypoxia-inducible factor-1α (HIF-1α) has been reported as closely associated with renal, liver and pulmonary fibrosis diseases. However, whether HIF-1α is involved in myocardial fibrosis, and the associations between HIF-1α, transforming growth factor-β_1_ (TGF-β_1_) and matrix metalloproteinase-9 (MMP-9) remain unknown. Therefore, this area warrants studying for the significance of AF diagnosis and treatment. The present study investigated the expression of HIF-1α in atrial fibrosis and its possible mechanism in isoproterenol (ISO)-induced rats. The three groups of rats; control, ISO and ISO plus sirolimus [also known as rapamycin (Rapa)], were treated for 15 days and sacrificed to remove the myocardial tissues. The expression levels of HIF-1α, TGF-β_1_ and MMP-9 and their associations with atrial fibrosis were examined through histomorphology and protein and mRNA levels. The protein and mRNA levels of HIF-1α, TGF-β_1_ and MMP-9 in the ISO group were increased markedly (P<0.01) compared with the control group, while those in the Rapa group were clearly decreased (P<0.01) compared with the ISO group. The protein and mRNA levels of HIF-1α, TGF-β_1_ and MMP-9 were positively correlated (P<0.01) with atrial fibrosis (collagen volume fraction index), as were the *HIF-1α*, *TGF-β_1_* and *MMP-9* mRNA levels (P<0.01) and the mRNA levels between *MMP-9* and *TGF-β_1_* (P<0.01). During the process of atrial fibrosis in ISO-induced rats, HIF-1α promotes the expression of TGF-β_1_ and MMP-9 protein, and thus is involved in in atrial fibrosis.

## Introduction

Clinically, atrial fibrillation (AF), which is of one of the most common types of arrhythmia, shows high disability and mortality rates in patients (1,2). In recent years, angiotensin II (AngII) and AF occurrence and maintenance has experienced increasing attention. The AngII levels in AF increase and eventually induce atrial fibrosis (3). Atrial fibrosis plays dual roles in inducing and maintaining AF (4–6). A previous study (7) showed that the expression level of hypoxia-inducible factor-1α (HIF-1α) is associated with AngII, which is involved in renal fibrosis. However, no associated study has been conducted for myocardial fibrosis. The present study refers to the method by Zhang *et al* (8), which used a subcutaneous bolus injection of isoproterenol (ISO) to induce AngII expression to establish an atrial fibrosis rat model. The HIF-1α inhibitor (9) [sirolimus, also known as rapamycin (Rapa)] was administered to examine the protein expression and mRNA levels of AngII, HIF-1α, transforming growth factor-beta_1_ (TGF-β_1_) and matrix metalloproteinase-9 (MMP-9) in myocardial tissue in the atrial fibrosis rat model, and thus the present study investigated their relevance and the possible mechanism of how HIF-1α following the ISO injection would induce atrial fibrosis.

## Materials and methods

### Animal model

Thirty healthy male Wistar rats, 180±20 g body weight, were purchased from the Lanzhou School of Medicine Animal Center in Lanzhou University (Lanzhou, China), and were maintained at 20–25°C with lighting-controlled circadian rhythms (8:00 am-10:00 pm) under normal feeding with free food and water. The rats were randomly divided into three groups of 10 rats: Control, ISO and ISO plus sirolimus (Rapa). The animal experiment was approved by the Animal Ethics Committee. The study referred to the method by Zhang *et al* (8) to establish an atrial fibrosis rat model. The ISO group rats were administered multipoint subcutaneous bolus injections of hydrochloric acid ISO (batch no. 080705; Shanghai Hefeng Pharmaceutical Co., Ltd., Shanghai, China), 5 mg/g/day, and once per day for seven days. The Rapa-intervention group rats were provided sirolimus oral solution (batch no. 110901; Hangzhou Zhongmei Huadong Pharmaceutical Co., Ltd., Hangzhou, China), specification 50 ml:50 mg, initiated on the second of the same ISO treatment as in the ISO group, 3 mg/kg/day (10), once per day and gavage for 14 days, with the interval between gavage and subcutaneous injection being 4–6 h. Simultaneously, the control and ISO groups were separately administered an equal amount of double-distilled water for stomach gavage as for the Rapa group. All the rats were sacrificed by cervical dislocation after 15 days.

### Sample collection and preservation

Along the coronary plane maximum transverse diameter, partial myocardial tissue was cut and placed in 10% formaldehyde solution for 24 h fixation. Following this, the tissue was paraffin-embedded and five serial slices (4-μm) were cut from it. Two slices were used for hematoxylin and eosin (HE) and Masson staining to observe the extent of myocardial fibrosis, which used collagen volume fraction (CVF) as the atrial fibrosis index. Immunohistochemistry (IHC) was performed on the remaining three slices to detect the expression of HIF-1α, TGF-β_1_ and MMP-9. The samples were obtained from the remaining cardiac tissue for detection of AngII by radioimmunoassay, and HIF-1α, TGF-β_1_ and MMP-9 expression levels by western blot (WB) analysis and reverse transcription quantitative polymerase chain reaction (RT-qPCR). The remaining cardiac tissue was cryopreserved in liquid nitrogen.

### Detection of AngII level in the myocardium in rats

A radioimmunoassay kit (Beijing North Institute of Biotechnology, Beijing, China) was used to detect the concentration of AngII.

### Observation of myocardial fibrosis

Myocardial tissue underwent formaldehyde fixation, dehydration, transparency, embedding in paraffin and slicing into two 4-μm sections. HE and Masson staining were subsequently performed, and the sections were mounted and observed by light microscope and radiography.

The CVF [CVF = (collagen area/total area of view field) × 100%] calculation was as follows: Three non-vascular vision images (magnification, ×400) were selected from each Masson staining slice. The image scanning software, Image-Pro Plus 6.0 (Media Cybernetics, Inc., Rockville, MD, USA), was used for image analysis and myocardial CVF calculation.

### IHC

Myocardial tissue paraffin sections underwent a number of steps, including dewaxing, antigen hot fix, blocking solution incubation, first and secondary antibody incubation, diaminobenzidine coloration, counterstaining, transparency and mounting. Phosphate-buffered saline (PBS) was used to replace the first antibody as the negative control, and the positive control was provided in the IHC kit (Boster Biological Tech Ltd., Wuhan, China). Rabbit anti-rat antibodies of HIF-1α, TGF-β_1_ and MMP-9 were all purchased from Santa Cruz Biotechnology, Inc. (Santa Cruz, CA, USA). The first antibody was diluted by 1:50, and the secondary goat anti-rabbit antibody was provided by Jackson ImmunoResearch Laboratories, Inc. (West Grove, PA, USA) under the dilution of 1:500.

### WB analysis

Myocardial tissue was placed in liquid nitrogen pre-cooling mortars, and a proper amount of protein lysate was added and centrifuged. Subsequent to removal of the supernatant, a small portion of the precipitation was used for the determination of protein concentration. Protein (100 μg) was mixed with 5× protein electrophoresis loading buffer, placed into a boiling water bath for 5 min, centrifuged at 12,000 × g for 1 min and fully loaded along with the protein marker (Fermentas, Waltham, MA, USA). In Tris-glycine buffer (pH 8.0) and under an 80 V voltage, a 1.5–2 h electrophoresis was performed, followed by a 20 V constant voltage and 1.5 h transferring to nitrocellulose film (Millipore, Billerica, MA, USA). The film was removed and 5% skimmed milk powder-sealed liquid/PBS with Tween (PBST) was added under room temperature and slow agitation was performed for 1.5 h. First antibody incubation was as follows: HIF-1α, TGF-β_1_ and MMP-9 antibodies were purchased from Santa Cruz Biotechnology, Inc., and diluted 1:300 in 5% skimmed milk powder/PBST at 4°C overnight; and anti-GAPDH (Santa Cruz Biotechnology, Inc.) monoclonal antibody was 1:10,000 diluted in 5% skimmed milk powder/PBST at 4°C overnight. PBST was used to wash the membrane three times for 10 min each. Secondary antibody incubation was performed as follows: Goat anti-rabbit antibody was 1:2,000 diluted in 5% skimmed milk powder/PBST and sheep anti-mouse (second for GAPDH) was 1:2,000 diluted in 5% skimmed milk powder/PBST, incubated at room temperature for 1 h, followed by washing of the membrane three times with PBST for 15 min each. The film was placed in the SuperSignal^™^ West Pico (Pierce, Rockford, IL, USA) for 2 min, tableted and developed to detect specific protein bands. The gel imaging system was photographed and the strip area and gray analysis of the protein zone was expressed by the integral gray value (D).

### RT-qPCR

TRIzol^®^ was added to 0.1 g myocardial tissue to extract RNA; 1–4 μg RNA was obtained for RT-qPCR. CFX-96 (Bio-Rad, Hercules, CA, USA) was used for fluorescence qPCR and the 20 μl PCR amplification reaction system was performed. Primer sequences were as follows: *HIF-1α* forward, 5′-ATCTCGGCGAAGCAAAGAGT-3′; reverse, 5′-TGACCA TCATCTGTTAGCACCAT-3′; *TGF-β_1_* forward, 5′-CTAATG GTGGACCGCAACAAC-3′; reverse, 5′-TAACGCCAGGAA TTGTTGCTAT-3′; *MMP-9* forward, 5′-CAAACCCTGCGT ATTTCCATT-3′, reverse, 5′-ACATCTCTCCTGCCGAGT TGC-3′; and *GAPD*H forward, 5′-AGTGCCAGCCTCGTC TCATAG-3′, reverse, 5′-CGTTGAACTTGC CGTGGGTAG-3′. *GAPDH* was used as the internal control. Each sample was loaded in three replicates. *HIF-1α*, *TGF-β_1_*, *MMP-9* and *GAPDH* RT-qPCR amplification conditions were as follows: Annealing temperature at 95°C for 15 sec, extension temperature at 60°C for 20 sec and melting curve temperature at 72°C for 20 sec, in a total of 45 cycles. At the end of the reaction, fluorescence quantitative data were collected including the amplification curve, working curve, melting curve and the corresponding Ct value, according to the 2^−ΔmRNAΔCt^ method to determine the relative mRNA expression level.

### Statistical methods

SPSS 17.0 software (SPSS, Inc., Chicago, IL, USA) was used for statistical analysis. All data are shown as mean±standard deviation. Single factor analysis of variance was used for inter-group comparison, least significant difference method was used for pairwise comparison, and the Pearson method was used for product-moment correlation analysis. P<0.05 was considered to indicate a statistically significant difference.

## Results

### Myocardial interstitial fibrosis

**Figure 1:**
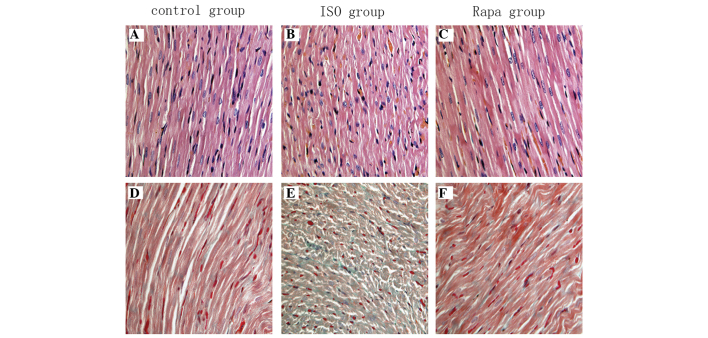
Myocardial interstitial fibrosis performance. *(*A–C) Hematoxylin and eosin staining; (D–F) Masson staining (green represents the myocardial interstitial collagen component). (A and D) Control; (B and E) ISO; and (C and F) Rapa groups (magnification, ×400). ISO, isoproterenol; Rapa, ISO plus sirolimus.

HE staining showed that the control group exhibited normal space between the myocardial nuclei, regular shape of the nuclei along the heart muscle and structured alignment of myocardial interstitial fibrosis (Fig. 1A). The ISO group had increased myocardial interstitial components and widened nuclear space. Fibrosis and cardiac muscle fiber shapes were disordered (Fig. 1B). The Rapa group showed a degree of reduction compared with the ISO group in the shape of the nuclei, and myocardial interstitial fibrous and interstitial fiber arrangements were disordered (Fig. 1C).

The Masson staining and CVF results showed that under light microscopy, normal myocardial interstitial collagen components appeared green (light green counterstained), the nuclei appeared blue, and myocardial fibers, cytoplasm and red blood cells appeared red (Fig. 1D-F). The image was analyzed to calculate the level of atrial fibrosis (CVF index), taking the average value as the measurement value. The control group (15.482±0.837%) did not show atrial fibrosis, and the Rapa group (16.730±1.052%) showed a greatly reduced atrial fibrosis level compared with the ISO group (86.704±1.982%) (P<0.01). The difference between the Rapa and control groups did not show a statistically significant difference (P>0.05).

### Ang II levels in the myocardium of rats by radioimmunoassay

The result showed that the ISO (139.402±4.431 ng/l) and Rapa (132.712±5.316 ng/l) groups had significantly increased Ang II levels (P<0.01) compared with the control group (31.172±7.271 ng/l).

### IHC

**Figure 2:**
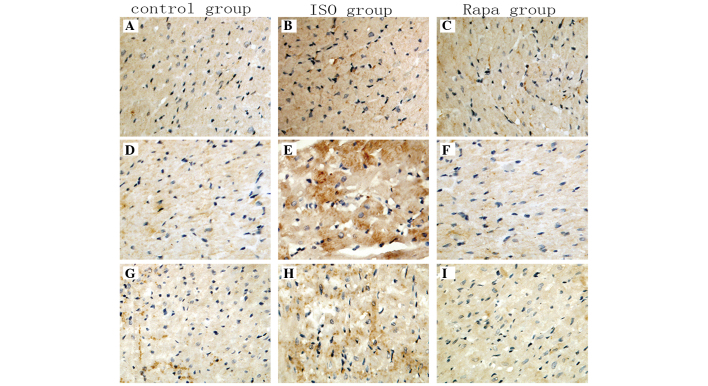
Immunohistochemistry results of HIF-1α, TGF-β_1_ and MMP-9 expression. Comparison of HIF-1α, TGF-β_1_ and MMP-9 expression in myocardial cells (claybank spots) in the cytoplasm represents positive staining. (A, D and G) Control; (B, E and H) ISO; and (C, F and I) Rapa groups (magnification, ×400). ISO, isoproterenol; Rapa, ISO plus sirolimus; HIF-1α, hypoxia-inducible factor-1α; TGF-β_1_, transforming growth factor-β_1_; MMP-9, matrix metalloproteinase-9.

Immunohistochemical detection of HIF-1α, TGF-β_1_ and MMP-9 expression showed claybank color for positive staining in myocardial cells of the rats under microscopy, distributed throughout the myocardial cytoplasm. The ISO group showed stronger expression levels than the control group, while the Rapa group showed markedly reduced levels of expression compared with the ISO group (Fig. 2).

### WB analysis and RT-qPCR

**Figure 3:**
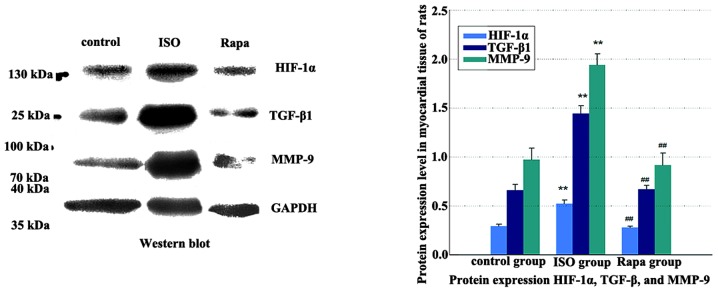
Protein expression of HIF-1α, TGF-β_1_ and MMP-9. ^**^P<0.01 compared with the control group, ^##^P<0.01 compared with the ISO group. ISO, isoproterenol; Rapa, ISO plus sirolimus; HIF-1α, hypoxia-inducible factor-1α; TGF-β_1_, transforming growth factor-β_1_; MMP-9, matrix metalloproteinase-9.

The *HIF-1α*, *TGF-β_1_* and *MMP-9* at the mRNA and protein levels were higher in the ISO group than those in the control group. The mRNA and protein expression in the Rapa group were significantly lower than those in the ISO group (Table I and Fig. 3).

### Associations among HIF-1α, TGF-β_1_ and MMP-9 expression levels in muscle fibrosis

In myocardial tissue, *HIF-1α*, *TGF- β_1_* and *MMP-9* mRNA expression levels were positively correlated (r=0.919, 0.997 and 0.985; P<0.01) with atrial fibrosis (CVF index). *HIF-1α*, *TGF-β_1_* and *MMP-9* mRNA expression levels themselves also exhibited a significant positive correlation (r=0.936 and 0.888; P<0.01). *MMP-9* and *TGF-β_1_* mRNA expression levels were positively correlated (r=0.981, P<0.01).

## Discussion

The majority of studies show that the renin-angiotensin system (RAS) is activated by AF, and simultaneously, as a major effecter molecule of the RAS in circulating and certain tissues (11), AngII levels increase and eventually induce atrial fibrosis (3). The present study identified that AngII levels in the ISO and Rapa groups in myocardial tissue were significantly higher than the control group after seven days of subcutaneous multiple high-dose continuous injection of ISO in rats, which implies that following ISO injection, RAS was activated and caused increased expression levels of AngII in myocardial tissue. According to the morphological observation, no atrial fibrosis was identified in the control group, while the ISO group had significant myocardial interstitial fibrosis, which indicates that high expression of AngII in myocardial tissues may be involved in atrial fibrosis formation, and this has been confirmed by a previous study (12).

The level of HIF-1α expression, as a tissue hypoxia index product, will increase during tissue hypoxia. Hypoxia has been linked to fibrosis (13), including in the liver (14,15), lung (16) and kidney (17). Although studies have shown that the increase in *HIF-1α* gene expression in the myocardium may be involved in its structural changes, including atrial fibrosis (18,19), no study has been conducted for the elevated AngII-induced myocardial fibrosis. The present study, from the aspects of pathology and protein and mRNA expression levels, found that the expression of HIF-1α in the ISO group was significantly higher than that in the control group, while when administering the HIF-1α inhibitor rapamycin intervention in the Rapa group, HIF-1α expression decreased significantly, and was positively correlated with myocardial fibrosis degree (CVF), which proves that AngII is involved in atrial fibrosis by regulating the expression of HIF-1α.

TGF-β_1_, as one of the AngII downstream factors (20), is associated with myocardial fibrosis occurrence (21). The present study also provides evidence for this. HIF-1α has a close association with TGF-β_1_ and can regulate its expression (22,23). The present study shows that in the ISO group, *TGF-β_1_* mRNA expression levels were much higher than those in the control group, and its expression in the Rapa group was significantly reduced. The expression of HIF-1α and the extent of myocardial fibrosis was positively correlated, which implies that HIF-1α can facilitate the expression level of TGF-β_1_ and thus induce atrial fibrosis.

In patients with AF, it has been reported that the atrial HIF-1α level rises with the increasing expression level of MMP-9 (24). MMP-9, as an important protease in the MMP family, is associated with myocardial matrix remodeling (25), and its increasing activity can result in acute myocardial fibrosis (26,27). The present study shows that in the ISO group, *HIF-1α* and *MMP-9* mRNA expression levels were significantly increased. They were positively correlated between themselves and also positively correlated with atrial fibrosis. Furthermore, HIF-1α can be involved in myocardial fibrosis formation by regulating the MMP-9 expression level. TGF-β_1_ is closely associated with MMP-9 and can regulate its gene level expression (28) and thus induce its synthesis (29). In the present study, it was also observed that in the ISO group, the *MMP-9* mRNA expression level was markedly higher than in the control group, while in the Rapa group this was significantly decreased. The expression level of TGF-β_1_ was also found to be positively associated with the degree of myocardial fibrosis. These results imply that TGF-β_1_ expression levels increase and cause the high expression of MMP-9, and thus aggravates myocardial fibrosis, which could be a possible mechanism in the AngII-induced atrial fibrosis model.

In conclusion, the present study shows that in the ISO-induced atrial fibrosis model, AngII, HIF-1α, TGF-β_1_ and MMP-9 were all highly expressed. By inhibiting the expression of HIF-1α, the expression levels of TGF-β_1_ and MMP-9 decreased accordingly, and the extent of myocardial fibrosis was also reduced. Considering the association among these factors, we can infer that, during the atrial fibrosis formation, HIF-1α promotes the expression of TGF-β_1_ and MMP-9 protein. A possible signal transduction pathway among HIF-1α, TGF-β_1_ and MMP-9 may exist, which could contribute significantly to the further study of the pathogenesis of AF and a new direction of drug research and development in AF therapy.

## Figures and Tables

**Table I:** Western blot analysis and RT-qPCR results of HIF-1α, TGF-β_1_ and MMP-9.

	HIF-1α		TGF-β_1_		MMP-9	
			
Group	Protein	mRNA	Protein	mRNA	Protein	mRNA
Control	0.294±0.021	(5.795±0.822)×10^−5^	0.660±0.059	(1.223±0.085)×10^−2^	0.974±0.116	(2.596±0.193)×10^−6^
ISO	0.522±0.039^a^	(2.103±0.492)×10^−4^^a^	1.444±0.081^a^	(7.511±0.156)×10^−2^^a^	1.939±0.113^a^	(4.854±0.210)×10^−6^^a^
Rapa	0.279±0.016^b^	(6.374±0.993)×10^−5^^b^	0.669±0.040^b^	(1.205±0.090)×10^−2^^b^	0.916±0.125^b^	(2.789±0.180)×10^−6^^b^

a P<0.01 compared with the control group;

b P<0.01 compared with the ISO group.

RT-qPCR, reverse transcription quantitative polymerase chain reaction; ISO, isoproterenol; Rapa, ISO plus sirolimus; HIF-1α, hypoxia-inducible factor-1α; TGF-β1, transforming growth factor-β1; MMP-9, matrix metalloproteinase-9.
